# Indirect Measurement of Rotor Dynamic Imbalance for Control Moment Gyroscopes via Gimbal Disturbance Observer

**DOI:** 10.3390/s18061873

**Published:** 2018-06-07

**Authors:** Liya Huang, Zhong Wu, Kan Wang

**Affiliations:** School of Instrumentation Science and Optoelectronics Engineering, Beihang University, Beijing 100191, China; liya_huang@buaa.edu.cn (L.H.); stjing@163.com (K.W.)

**Keywords:** control moment gyros, gimbal servo system, rotor imbalance, disturbance observer

## Abstract

The high-precision speed control of gimbal servo systems is the key to generating high-precision torque for control moment gyroscopes (CMGs) in spacecrafts. However, the control performance of gimbal servo systems may be degraded significantly by disturbances, especially a dynamic imbalance disturbance with the same frequency as the high-speed rotor. For assembled CMGs, it is very difficult to measure the rotor imbalance directly by using a dynamic balancing machine. In this paper, a gimbal disturbance observer is proposed to estimate the dynamic imbalance of the rotor assembled in the CMG. First, a third-order dynamical system is established to describe the disturbance dynamics of the gimbal servo system, in which the rotor dynamic imbalance torque along the gimbal axis and the other disturbances are modeled to be periodic and bounded, respectively. Then, the gimbal disturbance observer is designed for the third-order dynamical system by using the total disturbance as a virtual measurement. Since the virtual measurement is derived from the inverse dynamics of the gimbal servo system, the information of the rotor dynamic imbalance can be obtained indirectly only using the measurements of gimbal speed and three-phase currents. Semi-physical experimental results demonstrate the effectiveness of the observer by using a CMG simulator.

## 1. Introduction

As a kind of angular momentum exchange actuator, control moment gyros (CMGs) have been widely used in spacecraft attitude control owing to their superior properties in simple structure, large torque, and high precision [1,2,3,4,5]. A CMG typically consists of a high-speed rotor with large angular momentum and one or two low-speed gimbals [6,7]. According to torque instructions, the rotor can be rotated by gimbal motions driven by gimbal servo systems. With the variations of the direction of the rotor momentum, gyroscopic torque will be produced to control spacecraft attitude. In order to output high-precision torque to meet the need of spacecraft attitude control with high accuracy and stability, the control precision of gimbal servo systems should be high enough.

However, there exist many disturbances in the gimbal servo systems, such as friction, torque ripple, the disturbance torque induced by the rotor imbalance [8,9,10], etc. All these disturbances can deteriorate the control performance of gimbal servo systems significantly, especially the dynamic imbalance disturbance. With the amplitude proportional to the square of the rotor angular velocity and the same frequency as the rotor [8,11,12,13,14], it has been a great hindrance to the high-performance control of gimbal servo systems, since the high speed of the rotor will lead to severe disturbance with higher frequency and larger amplitude. Therefore, it is necessary to obtain the information regarding the rotor dynamic imbalance before designing the gimbal servo systems for CMGs.

Rotor mass imbalance is caused by the asymmetry of the rotor with respect to the spinning axis, owing to nonuniform mass distribution and imperfections in manufacturing [15,16,17]. Static imbalance results from the offset of the centroid from the spinning axis of the rotor. Dynamic imbalance results from the misalignment of the principal axis of inertia with respect to the spinning axis of the rotor. When the rotor rotates around the spinning axis, static and dynamic imbalance can produce radial centrifugal force and torque, respectively [18,19,20]. Since only the torque can rotate the gimbal, the dominant factor affecting gimbal servo systems is the dynamic imbalance, not the static one.

For a free rotor, the dynamic imbalance can be measured by using well-developed methods and then reduced with correction devices by adding or subtracting correction masses [21,22]. Owing to the limits of practical devices, a certain residual imbalance still exists in the rotor after dynamic balancing [23]. Furthermore, the dynamic imbalance may be changed by an error of assembly after the rotor is assembled on the mechanical bearing. In order to obtain the information of the dynamic imbalance for the assembled rotor, field dynamic balancing techniques can be used without disassembly [24,25]. However, these techniques are not suitable for the assembled CMGs owing to the effects of the gimbal motions.

For the measurement and suppression of the residual mass imbalance of magnetically suspended rotors installed in the equipment, various research results have been reported in literatures [26]. In one study [27], a disturbance observer was designed to estimate the matched disturbances, including imbalance, and then a composite control method was proposed to realize precision suspension of an active magnetic bearing (AMB) system. In another study [28], a repetitive disturbance observer-based controller was developed specially to reject the disturbance caused by the rotor mass imbalance of an AMB system. Another study [29] found that the lumped disturbances, including imbalance, were estimated by using disturbance equations and state measurements, and the vibrations were suppressed after disturbance compensation for electromagnetic actuators. However, these results are obtained by using the measurements of the rotor displacements which are only available in magnetically suspended rotors and not available in mechanical ones. Furthermore, the imbalance disturbance cannot be separated from the estimate of the total disturbance.

In order to obtain information about the dynamic imbalance for assembled rotors in CMGs, a gimbal disturbance observer is proposed in this paper. This observer is designed for a third-order system, describing dynamic imbalance disturbance and the other disturbances in the gimbal servo system. During the observer design, the total disturbance is regarded as a virtual measurement which can be achieved by using the inverse dynamics of the gimbal servo system. By using the gimbal disturbance observer, the dynamic imbalance disturbance can be separated from the total one, and the information of the rotor dynamic imbalance can be derived indirectly.

The rest of the paper is organized as follows. In Section 2, mathematical models for rotor imbalance and gimbal servo systems in CMGs are introduced, and the problem of the paper is formulated. In Section 3, the gimbal disturbance observer is designed for a third-order disturbance model, and the performance of the observer is analyzed by using the Lyapunov theory. In Section 4, a semi-physical experiment is carried out to verify the effectiveness of the observer. Finally, conclusions are given in Section 5.

## 2. Mathematical Model and Problem Formulation

### 2.1. Rotor Mass Imbalance

**Figure 1:**
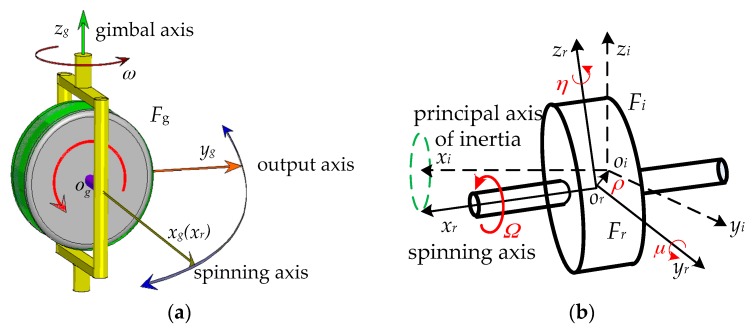
Schematics of SGCMG and rotor imbalance. (**a**) SGCMG; (**b**) Rotor imbalance.

The single gimbal CMG (SGCMG) is specifically considered in this paper, as shown in Figure 1a. A SGCMG is composed of a high-speed rigid rotor and a low-speed gimbal. Let the gimbal rotate about the gimbal axis *z_g_* at the velocity of *ω* and the rotor rotate about the spinning axis *x_g_* at the velocity of *Ω*, then SGCMG will generate gyroscopic torque along the output axis *y_g_* without consideration of installation errors, as follows:

(1) $$\tau =\omega I_{rx}\Omega$$

where *I_rx_* is the moment of inertia of the rotor about the spinning axis *x_g_*; axes *x_g_*, *y_g_*, and *z_g_* constitute an orthogonal gimbal frame *F_g_*(*o_g_x_g_y_g_z_g_*). Usually, *Ω* is fixed and high enough so that large gyroscopic torque can be achieved by using low gimbal speed. In order to realize high-accuracy and high-stability attitude control, the output torque of SGCMG should be accurate enough.

However, the output torque of SGCMG is inevitably disturbed by the rotor mass imbalance, owing to the uniform mass distribution and manufacturing tolerances of the rotor [21]. As shown in Figure 1b, static imbalance means that the centroid *o_i_* does not coincide with the geometric center *o_r_* of the rotor, while dynamic imbalance means that the principal axis of inertia *x_i_* does not coincide with the spinning axis *x_r_*. Two frames fixed to the rotor can be used to describe static and dynamic imbalance of the rotor, i.e., the geometric frame *F_r_*(*o_r_x_r_y_r_z_r_*) with the origin *o_r_* at the geometric center and axis *x_r_* along the spinning axis of the rotor, and the inertial frame *F_i_*(*o_i_x_i_y_i_z_i_*) with the origin *o_i_* at the centroid and coordinate axes along principal axes of inertia of the rotor. Static imbalance can be represented by the vector ***ρ*** from *o_r_* to *o_i_*, and dynamic imbalance can be represented by Euler rotation angles *μ* and *η* from *F_r_* to *F_i_*. When the rotor rotates at the velocity of *Ω*, static imbalance will lead to radial centrifugal force described in the gimbal frame by Luo et al. [17].

(2) $$\{\begin{array}{l} F_{sx}=0\\ F_{sy}=u_{s}\Omega^{2}\cos(\Omega t+\varphi_{s})\\ F_{sz}=u_{s}\Omega^{2}\sin(\Omega t+\varphi_{s}) \end{array}$$

where *u_s_* denotes the quantity of the rotor static mass imbalance and $\varphi_{s}$ denotes the initial phase of the static imbalance force in the gimbal frame. Dynamic imbalance will lead to radial centrifugal torque described in the gimbal frame by Luo et al. [17].

(3) $$\{\begin{array}{l} T_{dx}=0\\ T_{dy}=u_{d}\Omega^{2}\cos(\Omega t+\varphi_{d})\\ T_{dz}=u_{d}\Omega^{2}\sin(\Omega t+\varphi_{d}) \end{array}$$

where *u_d_* denotes the quantity of the rotor dynamic mass imbalance and $\varphi_{d}$ denotes the initial phase of the dynamic imbalance torque in the gimbal frame.

Both static and dynamic imbalance can cause vibrations in the spacecraft by transferring centrifugal force and torque from the rotor to the mounting base of SGCMG. Furthermore, the centrifugal torque produced by the dynamic imbalance can disturb the output torque and the gimbal motion of SGCMG. Once the control performance of the gimbal servo system is deteriorated by the dynamic imbalance torque, the accuracy of the output torque will be decreased again by the rotor dynamic imbalance.

### 2.2. Gimbal Servo System with Dynamic Imbalance Torque

The task of a gimbal servo system is to control the gimbal to rotate at a specific desired velocity to generate a desired torque for attitude control of the spacecraft. In most SGCMGs, a permanent magnet synchronous motor (PMSM) is utilized to drive the gimbal, owing to its superior performance at low speed. Along the gimbal axis, there are not only electromagnetic torque *T_e_* and internal disturbances *T_m_* produced by the motor, but also dynamic imbalance torque *T_dz_* produced by the rotor rotation and the gyroscopic torque *T_g_* arising from the spacecraft motion. Denoting the equivalent moment of inertia and the damping coefficient about the gimbal axis *z_g_* by *J* and *D* respectively, the equation of torque equilibrium about the gimbal axis can be written as

(4) $$J\dot{\omega }+D\omega =T_{e}+T_{m}+T_{dz}+T_{g}+T_{u}$$

where *T_u_* represents the unmodeled dynamics of the gimbal.

Assume that PMSM has a surface-mounted permanent magnet rotor, 3–phase wye-connected symmetric stator windings, and ideal back EMF of sinusoidal waveform, and then the electromagnetic torque can be written without consideration of salient and slot effects, magnetic saturation, and losses due to hysteresis and eddy current, as follows:

(5) $$T_{e}=-K_{T}[i_{a}\sin\theta_{e}+i_{b}\sin(\theta_{e}-120^{\circ })+i_{c}\sin(\theta_{e}+120^{\circ })]$$

where *i_a_*, *i_b_*, and *i_c_* are three-phase stator currents; *K_T_* and *θ_e_* denote the torque constant and the electrical angle of the motor respectively [30].

Although the proper electromagnetic torque *T_e_* can be produced to control gimbal motion by regulating three-phase stator currents *i_a_*, *i_b_*, and *i_c_* through velocity and current loops, the control performance is still affected by the disturbances in Equation (4) to a great extent. Compared with the other disturbances, the rotor dynamic imbalance torque has higher frequency and larger amplitude, and it has been a dominant factor in deteriorating the performance of the gimbal servo system. In order to design a gimbal servo system with high robustness to disturbances, the information of the rotor dynamic imbalance should be known in advance. However, it is very difficult to measure the dynamic imbalance of a rotor after it is assembled in a CMG.

### 2.3. Problem Formulation

The aim of this paper is to measure the rotor dynamic imbalance of a CMG indirectly from the information of the gimbal motion, and the problem of indirect measurement can be formulated as follows:

Design a gimbal disturbance observer for the CMG gimbal servo system to estimate the rotor dynamic imbalance torque *T_dz_* from Equation (4) by using the information of gimbal velocity *ω* and electromagnetic torque *T_e_*, and then calculate the quantity of the rotor dynamic imbalance according to Equation (3). Electromagnetic torque *T_e_* can be derived from Equation (5) according to the measurements of three-phase stator currents.

## 3. Indirect Measurement of Rotor Dynamic Imbalance

In this section, a third-order model is first established to describe the characteristics of the rotor dynamic imbalance disturbance and the other disturbances. Then, a gimbal disturbance observer is designed for the third-order disturbance model to estimate the rotor dynamic imbalance disturbance along the gimbal axis. Gain tuning guidelines and observer performance are also discussed in this section.

### 3.1. Disturbance Model

Denote the total disturbance in Equation (4) by *d*, i.e., *d = T_dz_* + *T_m_* + *T_g_* + *T_u_*, then Equation (4) can be rewritten as

(6) $$d=J\dot{\omega }+D\omega -T_{e}$$

Theoretically, the information of the total disturbance *d* can be obtained from Equation (6) if both angular velocity *ω* and electromagnetic torque *T_e_* are available. In practice, it is not feasible to obtain *d* by using Equation (6) since the calculation of the differential $\dot{\omega }$ will amplify higher-frequency noise components in *ω*. Even though the total disturbance *d* can be achieved, it is still difficult to obtain the information of the rotor dynamic imbalance torque *T_dz_*. In order to separate *T_dz_* from *d*, it is necessary to find characteristics that differentiate *T_dz_* from the other disturbances. From Equation (3), it is obvious that *T_dz_* is a sinusoidal function of time with a fixed angular frequency *Ω*, and the second-order derivative of *T_dz_* can be written as

(7) $${\ddot{T}}_{dz}=-u_{d}\Omega^{4}\sin(\Omega t+\varphi_{d})$$

According to Equations (3) and (7), the following equation can be obtained as

(8) $${\ddot{T}}_{dz}=-\Omega^{2}T_{dz}$$

Let *x*_1_ = *T_dz_*, *x*_2_ = ${\dot{T}}_{dz}$, *x*_3_ = *T_m_* + *T_g_* + *T_u_*, then a third-order state-space model can be derived from Equation (8) to describe the disturbance dynamics, as follows:

(9) $$\{\begin{array}{l} {\dot{x}}_{1}=x_{2}\\ {\dot{x}}_{2}=-\Omega^{2}x_{1}\\ {\dot{x}}_{3}=\delta (t) \end{array}$$

where *δ*(*t*) denotes the varying rate of *x*_3_.

Regard *d* in Equation (6) as a virtual measurement of the total disturbance, then the virtual output equation of System (9) can be written as

(10) $$d=x_{1}+x_{3}$$

Let $x=\left[\begin{array}{c} x_{1}\\ x_{2}\\ x_{3} \end{array}\right]$, $A=\left[\begin{array}{ccc} 0 & 1 & 0\\ -\Omega^{2} & 0 & 0\\ 0 & 0 & 0 \end{array}\right]$, $B=\left[\begin{array}{c} 0\\ 0\\ 1 \end{array}\right]$, $C^{T}=\left[\begin{array}{c} 1\\ 0\\ 1 \end{array}\right]$, then we can rewrite Equations (9) and (10) in a compact form as

(11) $$\{\begin{array}{l} \dot{x}=Ax+B\delta (t)\\ d=Cx \end{array}$$

Thus, the dynamics of the rotor dynamic imbalance disturbance and the other disturbances in the gimbal servo system can be described by Equation (11). According to Equation (11), the observation matrix can be expressed as

$$Q_{c}=\left[\begin{array}{c} C\\ CA\\ CA^{2} \end{array}\right]=\left[\begin{array}{ccc} 1 & 0 & 1\\ 0 & 1 & 0\\ -\Omega^{2} & 0 & 0 \end{array}\right]$$

Since rank (***Q***_c_) = 3, the pair (***A***,***C***) is observable; thus, a gimbal disturbance observer can be designed to estimate the disturbances.

### 3.2. Gimbal Disturbance Observer Design

In order to obtain the information of the rotor dynamic imbalance disturbance along the gimbal axis, a gimbal disturbance observer will be designed for Equation (11).

Let $\hat{x}$ and $\hat{d}$ represent the estimate of ***x*** and the prediction of *d*, respectively. According to the theory of Luenberger observer design, a state observer for Equation (11) can be designed as

(12) $$\{\begin{array}{l} \dot{\hat{x}}=A\hat{x}+L(d-\hat{d})\\ \hat{d}=C\hat{x} \end{array}$$

where *L* is the observer gain matrix with dimensions of 3 × 1.

**Figure 2:**
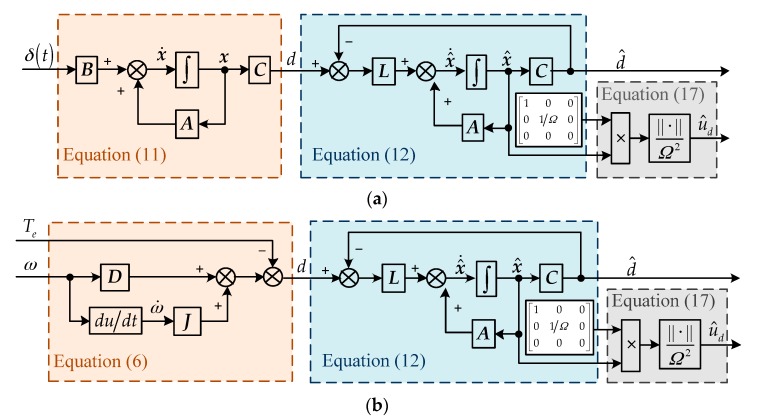

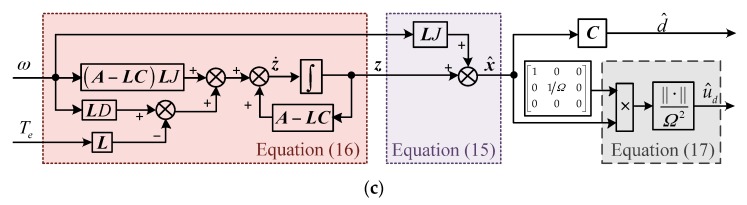
Design procedure of gimbal disturbance observer. (**a**) Observer design in theory; (**b**) observer with virtual measurement; (**c**) practical observer without the calculation of the differential $\dot{\omega }$.

The relationship between Equation (11) and Equation (12) is shown in Figure 2a. However, the output *d* in the observer (12) is only a virtual measurement, and it cannot be measured directly. In this paper, the information of *d* can be obtained indirectly from the system dynamics. According to the information of the angular velocity *ω* and electromagnetic torque *T_e_*, *d* can be calculated from Equation (6). By substituting Equation (6) into Equation (12), the observer with indirect measurement of *d* can be derived as

(13) $$\dot{\hat{x}}=A\hat{x}+L(J\dot{\omega }+D\omega -T_{e}-\hat{d})$$

Then the observer with indirect measurement of *d* can be plotted in Figure 2b. Since $\hat{d}=C\hat{x}$, Equation (13) can be rewritten as

(14) $$\dot{\hat{x}}=(A-LC)\hat{x}+L(J\dot{\omega }+D\omega -T_{e})$$

In order to avoid the calculation of $\dot{\omega }$ in Equation (14), an auxiliary variable *z* is introduced to satisfy

(15) $$\hat{x}=z+LJ\omega$$

By using the auxiliary variable ***z*** in Equation (15), the angular acceleration $\dot{\omega }$ can be eliminated in Equation (14), which can be changed into the following form as

(16) $$\dot{z}=\left(A-LC\right)z+\left(A-LC\right)LJ\omega +L\left(D\omega -T_{e}\right)$$

Thus, the gimbal disturbance observer can be formulated by Equations (15) and (16), and it is shown in Figure 2c. According to gimbal velocity *ω* and electromagnetic torque *T_e_*, the auxiliary variable ***z*** can be derived from Equation (16) and the estimate $\hat{x}$ can be derived from Equation (15) in turn. According to the definition of state variables *x*_1_ and *x*_2_, the quantity of the rotor dynamic imbalance can be estimated as

(17) $${\hat{u}}_{d}=\sqrt{{\hat{x}}_{1}^{2}+{\hat{x}}_{2}^{2}/\Omega^{2}}/\Omega^{2}$$

.

### 3.3. Observer Convergence Analysis

In order to analyze the convergence of the gimbal disturbance observer in Equations (15) and (16), the error dynamics of the observer should be obtained first. Since the observer in Equations (15) and (16) is obtained by the variable substitution of Equation (12), the error dynamics for Equation (12) can be examined instead.

By defining the estimation errors as $\tilde{x}=x-\hat{x}$ and $\tilde{d}=d-\hat{d}$, the error dynamics of the observer can be derived from Equations (11) and (12), as follows:

(18) $$\{\begin{array}{l} \dot{\tilde{x}}=(A-LC)\tilde{x}+B\delta (t)\\ \tilde{d}=C\tilde{x} \end{array}$$

Since the pair (***A***, ***C***) is observable, the matrix (***A*** − ***LC***) can be Hurwitz by choosing the suitable gain matrix ***L***. Then, given any matrix ***Q*** > 0, there must exist a unique matrix ***P*** > 0 such that

(19) $$P(A-LC)+{\left(A-LC\right)}^{T}P=-Q$$

For the error dynamics in Equation (18), choose the Lyapunov candidate function as

(20) $$V={\tilde{x}}^{T}P\tilde{x}>0$$

Taking the time derivative of Equation (20) along with Equations (18) and (19) gives

(21) $$\begin{array}{ll} \dot{V} & ={\dot{\tilde{x}}}^{T}P\tilde{x}+{\tilde{x}}^{T}P\dot{\tilde{x}}\\ & ={\tilde{x}}^{T}[P(A-LC)+{\left(A-LC\right)}^{T}P]\tilde{x}+2{\tilde{x}}^{T}PB\delta (t)\\ & =-{\tilde{x}}^{T}Q\tilde{x}+2{\tilde{x}}^{T}PB\delta (t) \end{array}$$

Let *λ*_1_, *λ*_2_, and *δ_m_* denote the minimal eigenvalue of ***Q***, the maximal eigenvalue of ***P***, and the upper bound of *δ*(*t*), respectively. Thus, Equation (21) can be enlarged as

(22) $$\begin{array}{ll} \dot{V} & \leq -\lambda_{1}{\left\|\tilde{x}\right\|}^{2}+2\left\|\tilde{x}\right\|\left\|P\right\|\left\|B\right\|\left|\delta (t)\right|\\ & \leq -\lambda_{1}{\left\|\tilde{x}\right\|}^{2}+2\lambda_{2}\delta_{m}\left\|\tilde{x}\right\| \end{array}$$

where ‖·‖ denotes 2-norm of a vector or matrix.

When $\left\|\tilde{x}\right\|>2\lambda_{2}\delta_{m}/\lambda_{1}$, we have $\dot{V}<0$ which will drive the trajectory of $\tilde{x}$ into a bounded region $R=\{\tilde{x}|\left\|\tilde{x}\right\|\leq 2\lambda_{2}\delta_{m}/\lambda_{1}\}$. The upper bound of ***R*** depends on *λ*_1_, *λ*_2_, and *δ_m_*, and it can be decreased by regulating the gain matrix ***L***.

### 3.4. Gain Tuning Guidelines

According to Equation (18), the transfer function from *δ*(*s*) to the disturbance estimation error $\tilde{d}(s)$ can be obtained as

(23) $$G(s)=\frac{\tilde{d}(s)}{\delta (s)}=C{\left[sI-(A-LC)\right]}^{-1}B$$

where ***I*** is an identity matrix with dimensions of 3 × 3.

Let ***L*** = [*l*_1_, *l*_2_, *l*_3_]^T^, then *G*(*s*) in Equation (23) can be rewritten as

(24) $$G(s)=\frac{s^{2}+\Omega^{2}}{s^{3}+(l_{1}+l_{3})s^{2}+(l_{2}+\Omega^{2})s+l_{3}\Omega^{2}}$$

From Equation (24), it is known that the characteristic polynomial of the error system in Equation (18) is as follows:

(25) $$D(s)=s^{3}+(l_{1}+l_{3})s^{2}+(l_{2}+\Omega^{2})s+l_{3}\Omega^{2}$$

If the bandwidth of Equation (18) is chosen as *λ* > 0, and the poles of Equation (24) are configured as −*λ*, −*λ ± j*Ω, then the characteristic polynomial can be written as

(26) $$D(s)=(s+\lambda )[{\left(s+\lambda \right)}^{2}+\Omega^{2}]=s^{3}+3\lambda s^{2}+(3\lambda^{2}+\Omega^{2})s+\lambda^{3}+\lambda \Omega^{2}$$

By comparing Equation (25) with Equation (26), the observer gains can be determined by

(27) $$\{\begin{array}{l} l_{1}=2\lambda -\lambda^{3}/\Omega^{2}\\ l_{2}=3\lambda^{2}\\ l_{3}=\lambda +\lambda^{3}/\Omega^{2} \end{array}$$

Thus, the problem of the observer gain tuning can be transformed into the choice of the bandwidth *λ* by using Equation (27). To demonstrate the effects of differences in bandwidth *λ* on the performance of the gimbal disturbance observer, the amplitude–frequency characteristics of *G*(*s*) can be used as an example.

**Figure 3:**
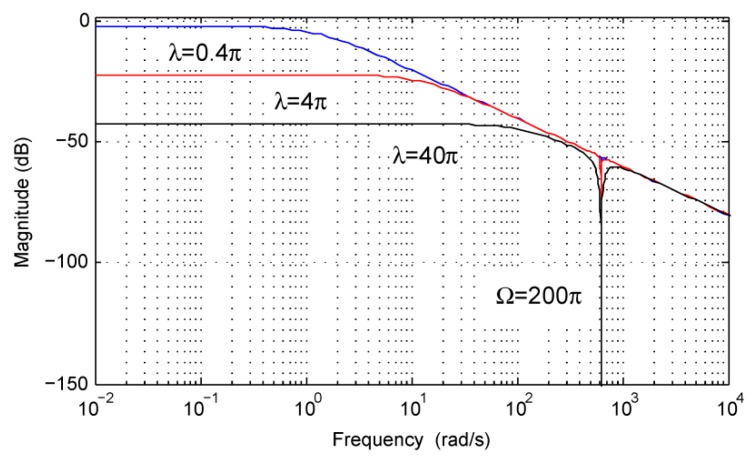
Amplitude–frequency characteristics of *G*(*s*).

By assuming the rotor velocity *Ω* = 200*π* rad/s, the amplitude-frequency characteristics of *G*(*s*) can be plotted in Figure 3 for *λ =* 0.4*π*, 4*π*, 40*π* rad/s. From Figure 3, it can be concluded that the disturbance *d* can be estimated precisely, even though the bandwidth *λ* is much less than the angular velocity *Ω* of the rotor. It is worth noting that the effect of the model uncertainty *δ*(*t*) can be more strongly attenuated with the rise of the bandwidth. However, a higher bandwidth can amplify noise components in the measurements of gimbal velocity *ω* and electromagnetic torque *T_e_*. The choice of the bandwidth *λ* is still a tradeoff between the attenuation ability of the model uncertainty and the measurement noise.

### 3.5. Discussions

According to the definition of ***L***, the disturbance observer in Equation (12) can be rewritten as

(28) $$\left\{ \begin{array}{l} {\dot{\hat{x}}}_{1}={\hat{x}}_{2}+l_{1}(d-\hat{d})\\ {\dot{\hat{x}}}_{2}=-\Omega^{2}{\hat{x}}_{1}+l_{2}(d-\hat{d})\\ {\dot{\hat{x}}}_{3}=l_{3}(d-\hat{d}) \end{array}\right.$$

Since $\hat{d}={\hat{x}}_{1}+{\hat{x}}_{3}$, Equation (28) can be rewritten as

(29) $$\left\{ \begin{array}{l} {\dot{\hat{x}}}_{1}={\hat{x}}_{2}+l_{1}(d-{\hat{x}}_{1}-{\hat{x}}_{3})\\ {\dot{\hat{x}}}_{2}=-\Omega^{2}{\hat{x}}_{1}+l_{2}(d-{\hat{x}}_{1}-{\hat{x}}_{3})\\ {\dot{\hat{x}}}_{3}=l_{3}(d-{\hat{x}}_{1}-{\hat{x}}_{3}) \end{array}\right.$$

Under zero initial conditions, the Laplace transformation of Equation (29) can be derived as

(30) $$\left\{ \begin{array}{l} s{\hat{x}}_{1}(s)={\hat{x}}_{2}(s)+l_{1}[d(s)-{\hat{x}}_{1}(s)-{\hat{x}}_{3}(s)]\\ s{\hat{x}}_{2}(s)=-\Omega^{2}{\hat{x}}_{1}(s)+l_{2}[d(s)-{\hat{x}}_{1}(s)-{\hat{x}}_{3}(s)]\\ s{\hat{x}}_{3}(s)=l_{3}[d(s)-{\hat{x}}_{1}(s)-{\hat{x}}_{3}(s)] \end{array}\right.$$

According to Equation (30), the transfer functions from the total disturbance *d* to ${\hat{x}}_{1}$ and ${\hat{x}}_{3}$ can be obtained as follows.

(31) $$\left\{ \begin{array}{l} G_{1}(s)=\frac{{\hat{x}}_{1}(s)}{d(s)}=\frac{l_{1}s^{2}+l_{2}s}{s^{3}+(l_{1}+l_{3})s^{2}+(l_{2}+\Omega^{2})s+l_{3}\Omega^{2}}\\ G_{3}(s)=\frac{{\hat{x}}_{3}(s)}{d(s)}=\frac{l_{3}(s^{2}+\Omega^{2})}{s^{3}+(l_{1}+l_{3})s^{2}+(l_{2}+\Omega^{2})s+l_{3}\Omega^{2}} \end{array}\right.$$

**Figure 4:**
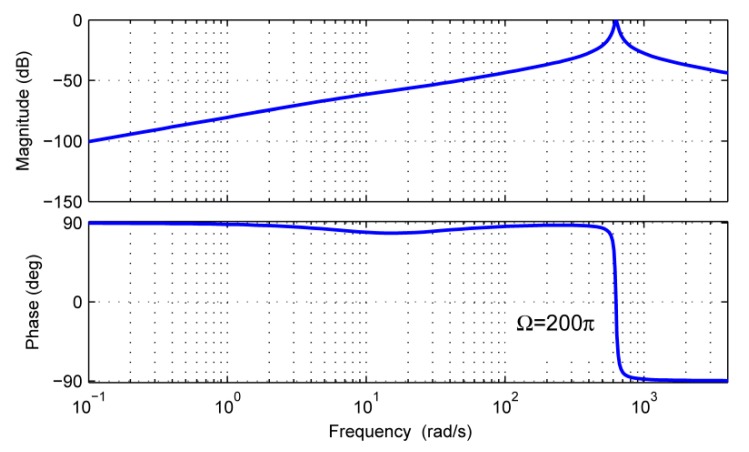
Frequency characteristics of *G*_1_(*s*).

**Figure 5:**
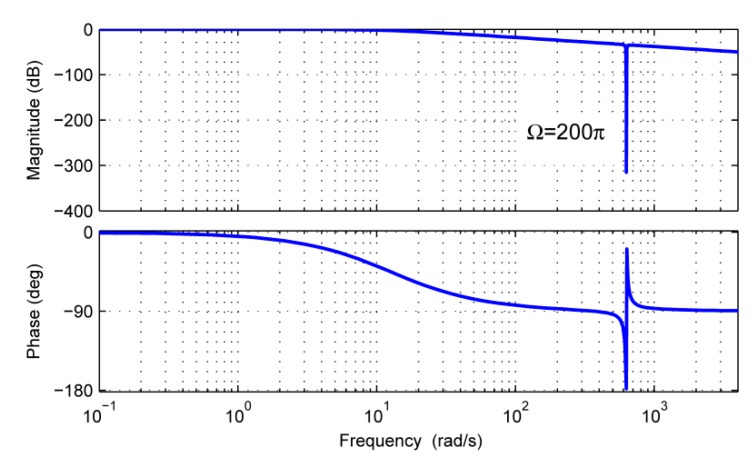
Frequency characteristics of *G*_3_(s).

In order to analyze the estimation performance of ${\hat{x}}_{1}$ and ${\hat{x}}_{3}$ in the frequency domain, we can take the observer bandwidth *λ =* 4*π* rad/s as an example. According to Equation (27), the observer gains *l*_1_, *l*_2_, and *l*_3_ will have definite values, and the frequency characteristics of *G*_1_(*s*) and *G*_3_(*s*) can be plotted in Figure 4 and Figure 5, respectively.

From Figure 4, it is obvious that the amplitude is $|G_{1}(j\Omega )|=1$ and the phase is $∠G_{1}(j\Omega )=0$ for the rotor imbalance frequency *Ω*. This means that the rotor dynamic imbalance disturbance in *d* can be completely transmitted to ${\hat{x}}_{1}$. While for the other components with the frequency much less or more than *Ω*, the transmission gains to ${\hat{x}}_{1}$ are greatly reduced. Therefore, ${\hat{x}}_{1}$ can realize the estimation of the rotor dynamic imbalance disturbance and the effects of the other frequency contents in *d* are very small.

From Figure 5, it can be seen that *G*_3_(s) has the characteristics of a low-pass filter. When the frequency contents are much lower than the bandwidth of 4*π* rad/s, they can be transmitted to ${\hat{x}}_{3}$. When the frequency contents are much higher than the bandwidth, the transmission gains to ${\hat{x}}_{3}$ are greatly reduced. Especially for the rotor imbalance frequency, the amplitude $|G_{3}(j\Omega )|=0$ and the imbalance disturbance in *d* cannot affect ${\hat{x}}_{3}$.

From the analysis of Figure 4 and Figure 5, it can be concluded that the rotor dynamic imbalance and the other disturbances in *d* can be estimated separately by using the gimbal disturbance observer. The rotor dynamic imbalance torque can be estimated by ${\hat{x}}_{1}$, and the other disturbances can be estimated by ${\hat{x}}_{3}$.

## 4. Experimental Results

**Figure 6:**
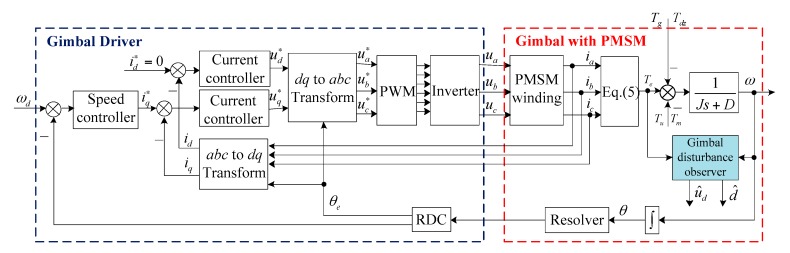
Block diagram of disturbance observer and gimbal servo system.

**Figure 7:**
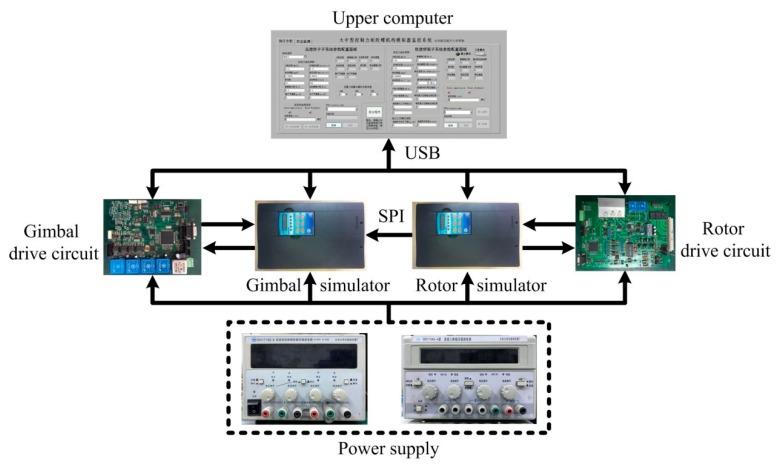
Structure of semi-physical experiment platform.

In order to verify the effectiveness of the gimbal disturbance observer, semi-physical experiments were carried out. The block diagram of the gimbal servo system and gimbal disturbance observer is shown in Figure 6, and the details of the gimbal disturbance observer are shown in Figure 2c. The semi-physical experiment platform included a CMG simulator, CMG drive circuits, an upper computer, and power supplies, as shown in Figure 7.

A CMG simulator is an electric load which has similar electrical and mechanical characteristics to practical CMG and has been used as a substitute for practical CMG to test CMG drive circuits in spacecraft engineering. It is composed of two parts, i.e., a gimbal simulator and a rotor simulator. The rotor simulator is in charge of simulating a brushless DC rotor motor with three switch-mode Hall position sensors, while the gimbal simulator is in charge of simulating a permanent magnet synchronous gimbal motor with two-channel resolvers. The rotor drive circuit is in charge of driving the rotor simulator, the information of which is transmitted to the gimbal simulator through serial peripheral interface (SPI). The gimbal drive circuit is in charge of driving the gimbal simulator, which can generate rotor dynamic imbalance torque and the other disturbances according to the rotor information and physical parameters. The physical parameters of the CMG simulator could be loaded by the upper computer, which also monitored the running states of the CMG simulator through USB. Since the actual value of the dynamic imbalance could be preset in the CMG simulator, it was very convenient to evaluate the performance of the indirect measurement method by comparing the estimated value with the preset one. When a practical CMG is used, the information of the actual value for the dynamic imbalance will be unavailable.

For the experiment, the preset parameters of the CMG simulator are listed in Table 1. The gimbal simulator was controlled to run by the gimbal drive circuit at the desired velocity of 1°/s, while the rotor simulator was controlled to run by the rotor drive circuit at 3000 r/min, 6000 r/min, and 9000 r/min, respectively. In the gimbal drive circuit, the sampling rate of the gimbal velocity *ω* and three-phase currents *i_a_*, *i_b_*, and *i_c_* were 5kHz. According to Equation (5), the electromagnetic torque *T_e_* can be derived from the sampled three-phase currents. By using the gimbal velocity *ω* and electromagnetic torque *T_e_*, the gimbal disturbance observer can be implemented with the bandwidth *λ* = 4*π* rad/s. Thus, the quantity of the rotor dynamic imbalance *u_d_* can be estimated from Equation (18) by using the estimated states of the gimbal disturbance observer. The internal disturbance *T_m_* is mainly caused by unexpected factors in gimbal servo systems, such as current ripples, flux distortion, and bearing friction. Since the gimbal simulator was controlled to run by the gimbal drive circuit, *T_m_* had already existed in the system, so we did not need to set the parameter of *T_m_* additionally. The gyroscopic torque produced by spacecraft motion was set to −0.06 Nm. The unmodeled dynamic *T_u_* also already existed in the system.

**Figure 8:**
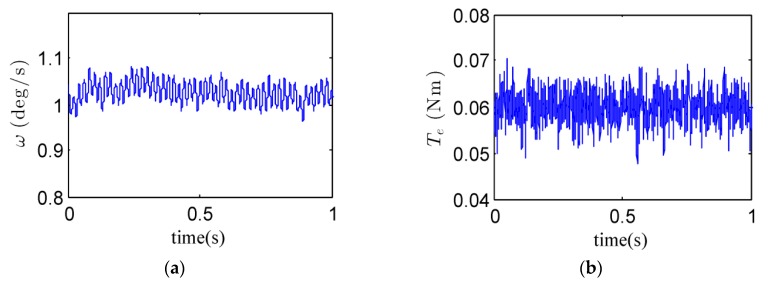

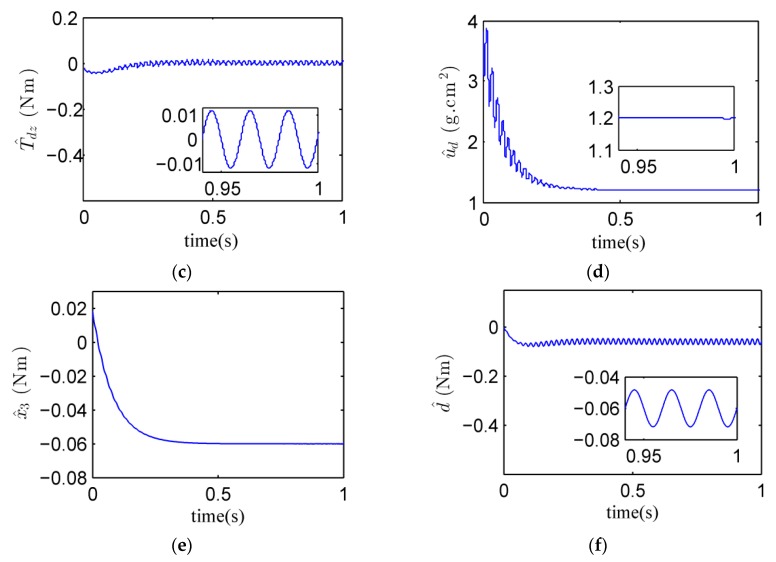
Experimental curves when *Ω* = 3000 r/min. (**a**) gimbal speed *ω*; (**b**) electromagnetic torque *T_e_;* (**c**) estimate of *T_dz_*; (**d**) estimate of *u_d_*; (**e**) estimate of *x*_3_; (**f**) estimate of *d*.

**Figure 9:**
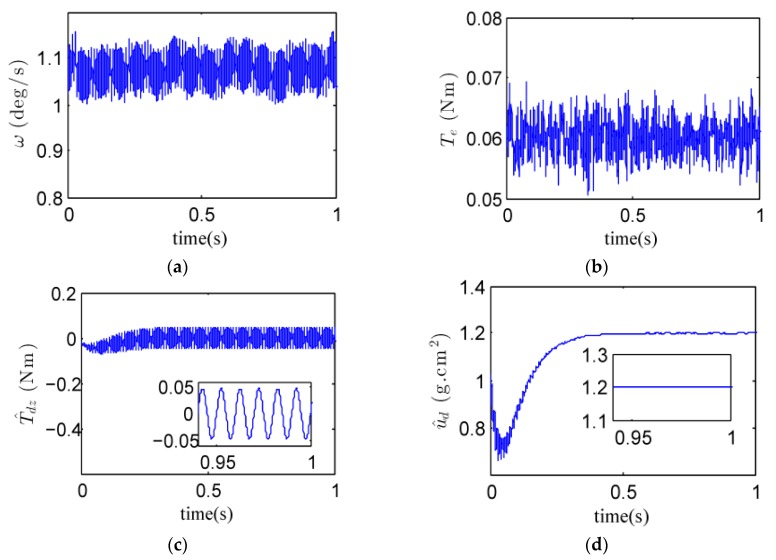

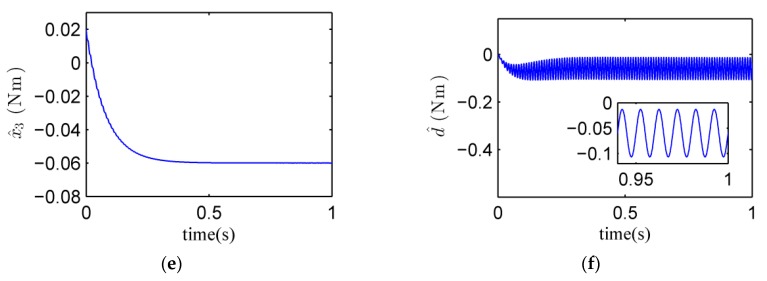
Experimental curves when *Ω* = 6000 r/min. (**a**) gimbal speed *ω*; (**b**) electromagnetic torque *T_e_;* (**c**) estimate of *T_dz_*; (**d**) estimate of *u_d_*; (**e**) estimate of *x*_3_; (**f**) estimate of *d*.

**Figure 10:**
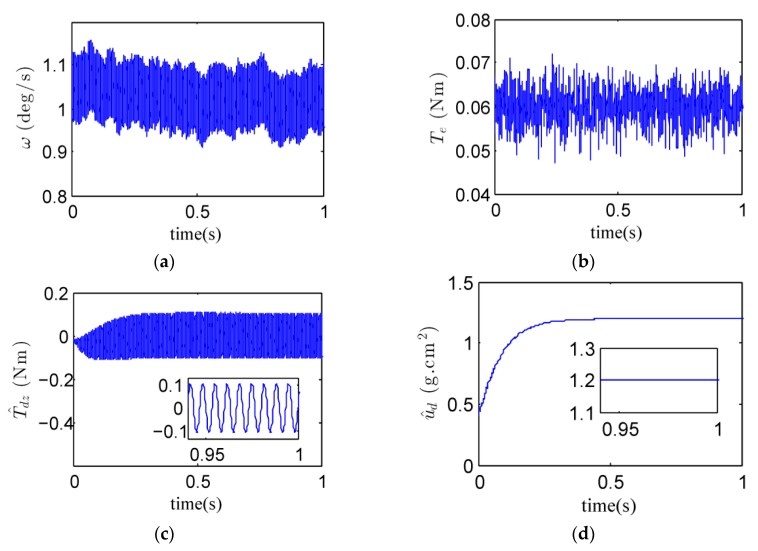

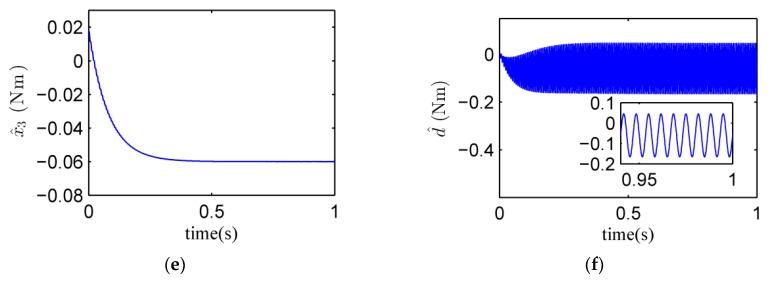
Experimental curves when *Ω* = 9000 r/min. (**a**) gimbal speed *ω*; (**b**) electromagnetic torque *T_e_;* (**c**) estimate of *T_dz_*; (**d**) estimate of *u_d_*; (**e**) estimate of *x*_3_; (**f**) estimate of *d*.

By using the above parameters and the observer bandwidth, experimental results for three different rotor velocities could be achieved, as shown in Figure 8, Figure 9 and Figure 10. Considering the transient process in the estimates of the quantity of the rotor dynamic imbalance, the mean of the estimates in the final 60 ms (i.e., from 0.94 s to 1 s) were taken as the final estimate. Additionally, the standard deviation (STD) was computed to evaluate the final estimate. Both the final estimate and the standard deviation are listed in Table 2.

(1) For the rotor speed of 3000 r/min, the experimental curves and the estimate of the quantity of the rotor dynamic imbalance are shown in Figure 8 and Table 2, respectively. From Figure 8a, it is obvious that the gimbal speed fluctuated periodically owing to the dynamic imbalance disturbance along the gimbal axis. From Figure 8c, it is known that the amplitude of the dynamic imbalance torque exceeded 0.01 Nm. From Figure 8d, it is known that the estimate of the quantity of the rotor dynamic imbalance is 1.1984 g·cm^2^ with the standard deviation of 0.0023 g·cm^2^. Figure 8e shows the estimate of the other disturbances. Since the internal disturbance torque *T_m_* in the gimbal motor was quite small, the main component of ${\hat{x}}_{3}$ is the estimate of the gyroscopic torque *T_g_* produced by spacecraft motion, which was set to −0.06 Nm. Figure 8f shows the estimate of the total disturbance *d*. It can be concluded from Figure 8c,e that the rotor dynamic imbalance and the other disturbances can be estimated separately by the proposed gimbal disturbance observer.

(2) For the rotor speed of 6000 r/min, the experimental curves and the estimate of the quantity of the rotor dynamic imbalance are shown in Figure 9 and Table 2, respectively. From Figure 9a,c, it can be seen that the gimbal speed fluctuated with many more ripples, since the dynamic imbalance disturbance had a larger amplitude and frequency than that in Figure 8. From Table 2, it is known that the estimate of the quantity of the rotor dynamic imbalance is 1.2003 g·cm^2^, and it is more accurate than the result derived from Figure 8. Figure 9e shows the estimate of the other disturbances *x*_3_, which is approximately −0.06 Nm, and Figure 9f shows the estimate of the total disturbance *d*.

(3) For the rotor speed of 9000 r/min, the experimental curves and the estimate of the quantity of the rotor dynamic imbalance are shown in Figure 10 and Table 2, respectively. From Figure 10a, it is clear that the fluctuations of the gimbal speed became heavier with the increase of the dynamic imbalance disturbance *T_dz_* in Figure 10c. The estimate of the quantity of the rotor dynamic imbalance in Table 2 is 1.1991 g·cm^2^ with smaller standard deviation. Figure 10e shows the estimate of the other disturbances *x*_3_. Similar to the first two cases, ${\hat{x}}_{3}$ is also about −0.06 Nm. Figure 10f shows the estimate of the total disturbance *d*.

From the above results, it can be concluded that the rotor dynamic imbalance and the other disturbances could be estimated successfully and separately by the gimbal disturbance observer in all three experimental cases. Comparatively, the estimates of the latter two cases have higher precision than that of the first one since the sampled information became richer with the increase of the rotor speed at the fixed sampling frequency.

## 5. Conclusions

The control performance of the CMG gimbal servo system degraded significantly by disturbances, especially the rotor dynamic imbalance disturbance with the same frequency as the high-speed rotor. This paper demonstrates that the total disturbance in a gimbal servo system can be described by a third-order dynamic model. Furthermore, a third-order gimbal disturbance observer is proposed to estimate the rotor dynamic imbalance and the other disturbances for an assembled CMG. By using the gimbal disturbance observer, the rotor dynamic imbalance disturbance can be precisely estimated and separated from the total one, and the quantity of the rotor dynamic imbalance can be estimated. In the observer, only the measurements of the gimbal speed and three-phase currents are required. Thus, the indirect measurement of the rotor dynamic imbalance can be realized. The effectiveness of the method has been verified by semi-physical experimental results.

## Figures and Tables

**Table 1:** CMG parameters.

Gimbal		Rotor	
Pole pairs	6	Pole pairs	8
Rated voltage	45 V (DC)	Rated voltage	45 V (DC)
Maximal speed	21 r/min	Maximal speed	10,000 r/min
Speed set point for test	1°/s	Speed set point for test	3000/6000/9000 r/min
Torque constant	1.435 Nm/A	Torque constant	0.035 Nm/A
Phase resistance	1.3 Ω	Phase resistance	0.5 Ω
Phase inductance	6.5 mH	Phase inductance	0.1 mH
Inertia	0.082 kg·m^2^	Inertia	0.039 kg·m^2^
Friction coefficient	0.001 Nm	Dynamic imbalance	1.2 g·cm^2^

**Table 2:** Estimates of rotor dynamic imbalance *u_d_*.

Rotor Speed	Mean Value	Standard Deviation
3000 rpm	1.1984 g·cm^2^	0.0023 g·cm^2^
6000 rpm	1.2003 g·cm^2^	0.0007 g·cm^2^
9000 rpm	1.1991 g·cm^2^	0.0006 g·cm^2^
